# Opioid Prescriptions in Chronic Pain Rehabilitation. A Prospective Study on the Prevalence and Association between Individual Patient Characteristics and Opioids

**DOI:** 10.3390/jcm10102130

**Published:** 2021-05-14

**Authors:** Mikael Svanberg, Britt-Marie Stålnacke, Patrick D. Quinn, Katja Boersma

**Affiliations:** 1 School of Health Sciences, Faculty of Medicine and Health, Örebro University, SE-701 85 Örebro, Sweden; 2 Centre for Clinical Research, Uppsala University, County Hospital, SE-721 89 Västerås, Sweden; 3 Department of Community Medicine and Rehabilitation, Rehabilitation Medicine, Umeå University, SE-901 87 Umeå, Sweden; britt-marie.stalnacke@umu.se; 4 Department of Applied Health Science, School of Public Health, Indiana University, Bloomington, IN 47405, USA; quinnp@iu.edu; 5 Center for Health and Medical Psychology (CHAMP), School of Law, Psychology and Social Work, Örebro University, SE-701 82 Örebro, Sweden; Katja.Boersma@oru.se

**Keywords:** chronic pain, rehabilitation, opioids, SQRP, biopsychosocial, interdisciplinary treatment

## Abstract

While against recommendations, long-term opioid therapy (LTOT) for chronic pain is common. This study aimed to describe the prevalence of opioid prescriptions and to study the association of patient characteristics (demographics, pain characteristics, anxiety, depressive symptoms and pain coping) with future LTOT. The sample included *N* = 1334 chronic musculoskeletal pain patients, aged 18–65, who were assessed for Interdisciplinary Multimodal Pain Rehabilitation (IMMR) in Swedish specialist rehabilitation. Prescriptions were tracked across a two-year target period after assessment. In total, 9100 opioid prescriptions were prescribed to 55% of the sample (M_median_ = 6, IQR = 14). Prediction of LTOT was analyzed separately for those who did (24%) and did not (76%) receive IMMR. The odds of receiving opioids was similar for these subsamples, after controlling for differences in baseline characteristics. In both samples, there were significant associations between patient characteristics and future opioid prescriptions. Dysfunctional pain coping was a unique predictor of LTOT in those who received IMMR while pain intensity and depressive symptoms were unique predictors in those who did not receive IMMR. The results underscore that opioid treatment is common among patients in chronic pain rehabilitation and relates to pain and psychological factors. Understanding in detail why these factors relate to opioid prescription patterns is an important future study area as it is a prerequisite for better management and fundamental for preventing overuse.

## 1. Introduction

Chronic pain is one of western societies’ largest public health problems [1]. About one in five European adults are affected, and chronic pain is one of the largest contributors (21%) to years lived with disability in the world [2,3]. Finding effective treatment for chronic pain is therefore crucial.

Interdisciplinary Multimodal Pain Rehabilitation Programs (IMMRPs) offer a well-coordinated interdisciplinary treatment conducted by a multidisciplinary team [4] and show significant benefits on a group level on perceived pain, function and return to work, compared with single treatment/unimodal rehabilitation [1,5]. IMMRPs are based on the biopsychosocial framework where the illness is understood as an interaction among biological, psychological and social factors [6]. IMMRPs are preceded by a multidisciplinary assessment that aims to assist in patient selection to the programs and development of a rehabilitation plan for those who will not participate, as well as to motivationally prepare the patient prior to IMMRP start [7]. This also includes an assessment and optimization of medication regimens which are thereafter held constant throughout program participation. IMMRPs are administrated over several weeks in group settings and support patients in the self-management of their condition and aim to optimize function. Based on cognitive and behavioral principles, they contain elements such as education, physical activation, coping skills training and occupational therapy sessions [1,8]. However, although there is a consensus that chronic pain should be treated within a biopsychosocial framework [1,6], long term treatment of chronic pain with opioids appears common [9,10] and there is reason for concern regarding its prevalence.

Although opioids are important tools for treating acute postoperative and traumatic pain, their comparative effectiveness and usefulness for chronic pain is questioned [11,12]. In brief, there are short-term effects on pain and function but a lack of evidence supporting longer-term (>four months) effects or superiority of opioids compared with other analgesics such as anti-inflammatory and anti-depressant drugs [13,14]. Opioid therapy and especially long-term use are also associated with serious adverse consequences such as side effects, risk for tolerance and hyperalgesia as well as misuse, dependency and depression [12,15,16]. Therefore, guidelines state that opioid prescriptions should be carefully thought through by physicians and, in cases of long-term use, gradual reductions should be considered [9,12,17]. Despite this, there is evidence that many patients with chronic pain continue to consume opioids beyond guideline recommendations. For example, a Swedish study of opioid prescription patterns showed that 27% of patients with chronic pain remained on opioids after three years [10]. This study also showed that 35% of these patients in addition were prescribed psychiatric medication (benzodiazepines and SSRI). Especially troublesome is the combination of opioids and benzodiazepines, as it is known to increase the risk of respiratory depression and fatal overdose [18].

Given that patients with chronic pain are frequently prescribed opioids while evidence and guidelines call for carefulness, it is important to increase our understanding of why patients receive long term prescriptions. This is a complex question where societal, systemic and contextual factors, attitudes and beliefs of prescribing physicians and patients, as well as how these interact in clinical encounters play a role (see for example [19,20]). This study focuses on the role of patient characteristics as patients with chronic pain often experience and express high levels of distress, helplessness and frustration in coping with their pain problem, which may partly steer physicians to issue opioid prescriptions. Several studies point to the importance of psychological factors. For example, general population studies show that preexisting mental health disorders are associated with increased likelihood of future opioid initiation and, especially, long-term opioid therapy (LTOT) [21,22]. In addition, patients’ own expectations about the likelihood of continuing regular opioid use have been found to predict long-term opioid use in patients with chronic pain who receive opioid therapy [23,24]. Indeed, co-morbidity of anxiety, depression and other psychosocial problems are common among patients receiving LTOT for chronic pain [17,25,26]. Moreover, patients with these risk factors may not only have an increased risk for LTOT but also of poor treatment response and harmful outcomes (e.g., misuse, opioid use disorder, overdose) [15,24,27,28]. However, while several studies indicate the potential significance of psychological factors in understanding the variation in LTOT among patients with chronic pain, the current state of research is based mostly on cross-sectional designs, each covering only a small selection of risk factors [17]. Furthermore, although there are studies showing that opioid prescriptions are common among patients with chronic pain, there are few studies detailing the prevalence of opioid prescriptions in the context of chronic pain rehabilitation. In this study, we therefore aim to describe the prevalence of opioid prescriptions and study the prospective association of individual patient characteristics with future opioid use. We use a biopsychosocial perspective, covering demographics, pain characteristics and psychological risk factors. We make use of a relatively large clinical sample of patients with chronic pain who underwent a multidisciplinary assessment in specialist rehabilitation care. The cohort was analyzed as two separate subsamples: those who do and do not participate in an IMMRP after assessment. We follow these two samples across a period of two years after initial assessment and track their opioid prescriptions. Specifically, we aim to study:

1. The prevalence of opioid prescriptions and whether there are any differences between subsamples in opioid prescription prevalence.
2. The associations of opioid prescriptions with patient characteristics (specifically demographics, pain characteristics, anxiety and depressive symptoms and pain coping profiles) within each sample.
3. The predictive value of patient characteristics for LTOT within each sample.

## 2. Materials and Methods

### 2.1. Design

This is a prospective cohort study analyzing the relation between patient reported data collected during multidisciplinary assessment and individual opioid prescription data collected during a two-year period after this assessment. The study was granted ethical clearance by the Umeå University Ethics Committee (D-nr: 2013/192-31 and 2017/438-32).

### 2.2. Subjects and Setting

Subjects are consecutive patients with chronic pain, who were referred, mostly from primary care, to clinical rehabilitation departments at two university hospitals in the north and the middle of Sweden between 2008 and 2012. Subjects were patients in regular health care service, they were assessed and received treatment on an outpatient basis. Inclusion criteria for the IMMRP were disabling chronic pain, age between 18 and 65 years, no further medical investigations needed, written consent to participate and agreement not to participate in other parallel treatments. Exclusion criteria were ongoing major somatic or psychiatric disease, a history of significant substance abuse and state of acute crisis.

From a target sample of *N* = 1624 patients, *N* = 1334 (82%; Mage = 40.9; SD = 11.3) patients could be included in the analytical sample. *N* = 290 (17.8%) patients were excluded because of missing data on target independent variables. The patients who were excluded did not differ from the analytical sample in terms of age, pain intensity, pain coping or anxiety symptoms. There were however small but significant differences in education (post-upper-secondary education: excluded sample, 16%; analytical sample, 22%; χ^2^ (1) = 4.61, *p* = 0.03), spreading of pain (number of pain regions: excluded sample, M = 12.7, Sd = 9.4; analytical sample, M = 14.0, Sd = 8.1; t(387.78) = −2.22, *p* = 0.03), sex (women: excluded sample, 67%; analytical sample, 74%; χ^2^ (1) = 4.73, *p* = 0.03), depressive symptoms (excluded sample, M = 8.9, Sd = 4.8; analytical sample, M = 8.1, Sd = 4.5; t(1574) = 2.35, *p* = 0.02), IMMRP participation (excluded sample, 17%; analytical sample, 24%; χ^2^ (1) = 7.65, *p* = 0.006) and prescribed opioids one year before assessment (excluded sample, 46%; analytical sample, 39%; χ^2^ (1) = 4.46, *p* = 0.02).

### 2.3. Measures

#### 2.3.1. Data Collection

The patients filled out a paper and pencil questionnaire with instruments included in the Swedish Quality Registry for Pain rehabilitation (SQRP) [29] as part of a multidisciplinary assessment (96% of all consecutive patients). The data were thereafter transferred to the SQRP electronic database [30]. Nationally, approximately 98% of all referred patients deliver data to this register.

#### 2.3.2. Demographic Variables

Assessed demographics included age, sex and education (% post upper secondary education, for example college and university education).

#### 2.3.3. Pain Characteristics

Assessed pain characteristics included pain intensity and number of pain locations. Pain intensity was assessed with a numerical pain rating scale, asking patients to rate average pain during the past week on a scale of 0–10 with endpoints ‘no pain’ and ‘worst possible pain’. The number of pain locations was assessed using 36 predefined anatomical areas (18 on the front and 18 on the back of the body). Areas were summed to form a total score ranging from 0–36.

#### 2.3.4. Depressive and Anxiety Symptoms

The Hospital Anxiety and Depression Scale (HADS) [31] was used to measure depressive and anxiety symptoms. The 14 items are rated on 4-point numerical scales (end points varying with the statement) and summed into 7-item depression and anxiety subscales (HAD-D and HAD-A; range 0–21). HADS-D and HADS-A have been found to have good psychometric characteristics [32,33].

#### 2.3.5. Pain Coping

Pain coping was assessed using pain coping classifications of the (WHY) MPI. The (WHY) MPI is a psychometrically sound, 61-item self-report questionnaire measuring psychosocial, cognitive and behavioral correlates of chronic pain [34,35]. Through established algorithms, the (WHY) MPI also identifies pain coping profiles, specifically the degree to which patients can be classified as displaying adaptive pain coping, dysfunctional pain coping and interpersonally distressed pain coping. An adaptive coping profile is characterized by low pain severity, low pain interference, low affective distress, high perception of life control and high activity level. A dysfunctional coping profile is characterized by high pain severity, high interference, high affective distress, low life control and low activity level. An interpersonally distressed coping profile is characterized by lower levels of perceived support from significant others and high degree of pain affective distress. The degree to which patients match each coping profile is translated to a value on a 0–100 scale [36]. The reliability and validity of these coping profiles has been replicated in several studies in different countries, including in Sweden [37,38]. In this study, we use the dysfunctional and interpersonally distressed coping classifications. The adaptive coping profile was excluded as it conceptually mirrors the opposite of the dysfunctional coping scale and was therefore considered redundant.

### 2.4. Participation in IMMRP

All *N* = 1334 patients received assessment by a physician and typically also a psychologist, physiotherapist, social worker and occupational therapist. *N* = 321 (24%) also participated in an IMMRP. Participation in an IMMRP is dependent on the multidisciplinary assessment outcome (e.g., no other medical investigations needed; professional judgement that the pain condition can be improved by multidisciplinary support in pain management) as well as on whether the patients themselves consent to participate and agree not to engage in other parallel treatments [8,39]. The IMMRP was conducted in groups of 6–9 participants and lasted for about one to two months. The patients were trained in pain management strategies and had physical and ergonomic training under supervision of physiotherapists and occupational therapists. There were broad and general goals such as improved activity levels and life satisfaction, in combination with individualized goals of the patient. Details for the IMMRP content, the inclusion and exclusion criteria have been described in more detail elsewhere [39]. If the assessment by the multidisciplinary team considered that the patient needed further investigation or did not fulfil the inclusion criteria for the IMMRP, a rehabilitation plan was presented to the patient and their general practitioner with suggestions and recommendations for further treatment. All patients were referred back to primary care after the assessment if they did not participate in IMMRP and after the IMMRP if they did.

### 2.5. Opioid Prescriptions

Opioid prescriptions were extracted from the Swedish Prescribed Drug Register across a window of one year before (to control for prior use), and two years after (the target period) the year during which assessment took place. This target period was chosen as it extends beyond the IMMRP (duration three to four months) and its follow-up period (1 year) while at the same time retaining a proximity to time of baseline assessment and IMMRP that may reasonably affect prescription patterns. The Swedish Prescribed Drug Register includes prescription and dispensing dates and information on products and prescribers for all Swedish dispensed prescription medications [40]. The register is under strict confidentiality but is accessible for research upon request to the National Board of Health and Welfare after ethical clearance.

In line to Swedish rules and regulations, and after ethical clearance, data from the SQRP was delivered to independent data officials at the Swedish National Board of Health and Welfare who conducted the data linkage with The Swedish Prescribed Drug Register. SQRP data were linked with prescription records on an individual basis, using Swedish person identification numbers. Data were thereafter de-identified and subsequently delivered back to the research team.

Anatomical Therapeutic Chemical (ATC) codes were used to identify opioids prescribed between 1 July 2005 and 31 December 2017. In line with previous studies, we did not include opioids used for intravenous distribution, treatment of Substance Use Disorder (SUD) or other diagnoses that were not relevant for the aim of this study, e.g., cough [21]. For specifications of in- and excluded ATC codes, see Table S1.

Opioid prescriptions were operationalized as follows: (1). Whether opioids were prescribed during the two-year target period: yes/no; (2). Whether opioids were classified as weak (i.e., codeine, dextropropoxyphene and tramadol) or strong opioids. (3). Whether patients had long-term opioid therapy (LTOT). There is not one agreed upon definition of LTOT, but, as in Sweden opioids are prescribed for a maximum of 3 months’ supply [41], we defined LTOT as the prescription of strong opioids (ATC code N02A group II, IV) during three of four consecutive 3-month quarters during the 2-year period after multidisciplinary assessment. This is in line with several studies [42,43]. In this study, prescription dates were used, but all prescribed opioids recorded in the study were also dispensed from pharmacies.

### 2.6. Statistical Analysis

All statistics were performed in SPSS (version 24.0). As assignment to IMMRP participation was non-random, the cohort was analyzed as two subsamples: those who do and do not participate in an IMMRP after assessment. T-tests and Chi-squared tests were used to study baseline and opioid prescription differences between the two samples. Point-biserial and Phi correlations were used to study univariate associations between baseline patient characteristics and opioid prescriptions within each sample. Logistic regressions were used to model multivariate associations between predictors (baseline patient characteristics showing significant univariate associations with LTOT) and LTOT within each sample. In all predictive models, assessment location was controlled for. Analyses were performed with, and without, controlling for strong opioids prescribed during the year prior to assessment.

## 3. Results

### 3.1. Overall Prevalence of Opioid Prescriptions

The overall prevalence and distribution of different kinds of opioids for the total sample are displayed in Table 1. A total of 9100 opioid prescriptions were prescribed to *N* = 741 (55%) of the 1334 patients (M_median_ = 6, IQR = 14) during the two-year target period. The proportion of strong opioids was 31%. The most common weak opioids prescribed were tramadol (46.9%) and codeine/paracetamol (19.8%). The most common strong opioids prescribed were oxycodone (11.3%) and morphine (7.5%).

### 3.2. Baseline Characteristics of Subsamples

Table 2 shows baseline characteristics of the samples participating and not participating in IMMRP. At the time of the multidisciplinary assessment, there were no statistically significant differences between those who did and did not continue to the IMMRP in level of education, pain intensity, spreading of pain, interpersonally distressed pain coping, dysfunctional pain coping, anxiety or depressive symptoms and whether they had been prescribed opioids during the year prior to assessment. There were significant differences in sex (IMMRP sample, 84% women; no IMMRP sample 70% women; χ^2^ (1) = 23.29, *p* < 0.001), age (IMMRP sample, *M* = 38.7, (Sd = 9.9); no IMMRP sample, *M* = 41.6, (Sd = 11.6); t(1332) = 4.10, *p* < 0.001) and the proportion of the sample who were prescribed strong opioids during the year prior to assessment (IMMRPs sample, 3.1%; no IMMRP sample, 8.1%; χ^2^ (1) = 9.41, *p* = 0.002).

### 3.3. Similarities and Differences in Opioid Prescription Prevalence between Subsamples

Table 2 displays the prevalence of opioid prescriptions for the two samples during the two-year target period. The overall distribution of opioid prescriptions was similar for those who did and did not participate in the IMMRPs, but the proportion of strong opioid prescriptions within the samples differed. Specifically, among those who did participate in the IMMRPs, there were *N* = 40 (12.5%) patients who received a strong opioid prescription whereas there were *N* = 203 (20%) who received a strong opioid prescription among those who did not participate in the IMMRP. This is a significant difference (χ^2^ (1) = 9.40, *p* = 0.002) but the odds of receiving a strong opioid prescription among those who did not participate in the IMMRP was not significantly higher after controlling for baseline differences between samples (age, sex, strong opioid prescriptions in the year prior to assessment; B = 0.34, SE = 0.20; OR = 1.40 (0.95–2.05), *p* = 0.09).

A total of *N* = 77 patients (6%) received LTOT during the two-year target period. The distribution of LTOT was different for those who did and did not participate in the IMMRP. Specifically, among those who did participate in the IMMRP, there were *N* = 10 (3%) patients who received LTOT while there were *N* = 67 (7%) who received LTOT among those who did not participate in the IMMRP. This is a significant difference (χ^2^ (1) = 5.43, *p* = 0.02) but the odds of receiving LTOT among those who did not participate in the IMMRP was, although coming close, not significantly higher after controlling for baseline differences between samples (age, sex, strong opioid prescriptions in the year prior to assessment; B = 0.49, SE = 0.26; OR = 1.40 (0.99–2.68), *p* = 0.06).

### 3.4. Associations of Baseline Patient Characteristics with Future Opioid Prescriptions

Table 3 displays correlations between demographics, pain characteristics, pain coping scores, depressive and anxiety symptoms, prior opioid prescriptions and opioid prescriptions during the two-year follow up period, in the samples receiving IMMRP and no IMMRP, respectively.

In the sample who participated in the IMMRP, receiving a prescription of at least one opioid (any) during follow-up was significantly associated with lower education, less spread pain, more dysfunctional coping, less interpersonally distressed coping and having been prescribed an opioid (any) during the year prior to assessment. Receiving at least one strong opioid prescription during follow up was significantly associated with sex (male), more dysfunctional coping and having been prescribed a strong opioid during the year prior to assessment. Receiving LTOT during follow up was significantly associated with more spread pain, more dysfunctional coping and having been prescribed an opioid (any or strong) one year prior to assessment.

In the sample who did not participate in IMMRP, receiving a prescription of at least one opioid (any) during follow up was significantly associated with lower education, more pain, more spread pain, more dysfunctional coping, less interpersonally distressed coping, more depressive symptoms and having been prescribed an opioid (any or strong) the year prior to assessment. Receiving at least one strong opioid prescription during follow-up was significantly associated with age (being older), more pain, more dysfunctional coping, more depressive symptoms and having been prescribed an opioid (any or strong) one year prior to assessment. Finally, receiving LTOT during follow up was significantly associated with more pain, more dysfunctional coping, more depressive symptoms and having been prescribed an opioid (any or strong) one year prior to assessment.

### 3.5. Predictive Value of Baseline Patient Characteristics for Future Long-Term Opioid Use (LTOT)

Table 4 and Table 5 show the results of the logistic regressions predicting the likelihood of receiving LTOT, in the samples receiving IMMRP and no IMMRP, respectively. The variables showing a univariate association to LTOT (see Table 3) were entered in the analyses. In the sample participating in the IMMRP, lower pain spreading was a significant predictor of LTOT while dysfunctional coping did not reach significance. However, when controlling for whether patients had been prescribed a strong opioid during the year prior to assessment, the analysis showed that dysfunctional pain coping was a significant predictor of LTOT while pain spreading did not reach significance. Note however, that while the *p*-values shifted, the odds ratios for these predictors were unchanged.

In the sample who did not participate in the IMMRP, the analysis showed that pain intensity and depressive symptoms were significant predictors of LTOT, irrespective of whether strong opioid prescription in the year prior to assessment was controlled for. Dysfunctional pain coping was not significant in these analyses.

## 4. Discussion

### 4.1. Key Results and Interpretation

In this prospective cohort study on patients with chronic pain, we found that more than half (55%) of the participants received opioids during the two years after a multidisciplinary assessment in specialist rehabilitation care. For those who received opioids, the median number of prescriptions was six, and of all opioids prescribed a third were strong opioids. The sample who, after assessment, did not participate in the Multidisciplinary Rehabilitation Program (IMMRP) had a higher likelihood of future prescription of strong opioids and long-term opioid therapy (LTOT) compared to those who did participate in the IMMRP. However, this increased likelihood was non-significant after controlling for differences in baseline characteristics. Specifically, patients who did not participate in the IMMRP were more likely to be male, were older and had higher degree of strong opioid use prior to assessment. Non-significance after controlling for these variables suggests that the association of IMMRP participation with opioid prescription may be accounted for by these patient characteristics. However, as the *p*-value was close to significance (*p* = 0.09 for strong opioids, *p* = 0.06 for LTOT), and prevalence of strong opioid use and LTOT was relatively low, it could be that IMMRP participation itself is related to future opioid use. Future studies, making use of the whole Swedish Quality Registry for Pain rehabilitation (including over 50,000 patients) could focus on the effect of IMMRP participation on opioid use. Not only would larger numbers ensure sufficient power, such studies could use methods such as propensity score matching to maximize comparability between those who do and do not participate in IMMRP after assessment.

The apparent interrelation between patient demographic characteristics, opioid prescription history and choice of pain treatment raises the important question of whether the prescription of strong opioids may discourage patients from engaging with non-pharmacologic (or at least non-opioid-intensive), self-management-oriented treatments like the IMMRP. While this study cannot provide a definite answer to this question, within group analyses of the sample not receiving IMMRP showed that there is a consistent association between opioid prescription history, pain, depressive symptoms, dysfunctional pain coping and future opioid prescriptions. In a previous study on this same sample, we found that non-IMMRP participation was also related to higher levels of previous healthcare seeking, pain-related fear as well as with a lower level of pain acceptance [44].

One possible explanation for these associations may be that these patients have gotten caught up in so called ‘misdirected problem solving’ [45]. The misdirected problem solving model is based on the well-known fear-avoidance model, which stresses the key role of pain-related distress and avoidance in the development of chronic pain problems [46]. In addition, the misdirected problem solving model underscores how patient worries and beliefs are embedded in a larger sociomedical context where biomedically oriented problem solving is reinforced and interacts with patients distress and helplessness in coping with their pain problem. The model highlights that the individual struggle to make sense of, and deal with, chronic pain interacts with a cultural and medical context that reinforces a sustained somatic focus and a continuous opting for biomedical interventions instead of psychosocial self-management [20]. Indeed, it has been found that the likelihood of biomedical intervention in clinical encounters is positively related to the degree to which patients elaborate on their somatic symptoms and inversely related to patients’ and doctors psychosocial talk [47,48]. In addition, it has been found that, besides somatic symptom elaboration, patients do indeed provide cues to the social and psychological dimensions of their suffering. However, what has been shown is that in general, physicians did not engage with these cues [49]. Thus, it may be that opioid therapy is not so much the result of patient demand or of physicians’ eagerness to prescribe, but rather the result of a doctor’s response to a patient’s elaboration of their pain symptoms and a lack of response to emotional and psychosocial cues, to which physicians may not have any well-trained clinical response available. There is a dire need for future studies that try to unravel the interpersonal dynamics of why patients increase their symptom presentation and the motivations behind why doctors in turn respond to this by providing opioid prescriptions.

One main finding of this study is that, besides pain, depressive symptoms were a unique predictor of future long-term opioid therapy (LTOT) for those not receiving the IMMRP. This is in line with previous research where mental health disorders are common in patients with LTOT [9,24]. While dysfunctional coping was associated to opioid outcomes, it was not unique predictor over and above pain and depressive levels. However, as argued above, it may be important in order to understand patients’ pain psychology. The unique predictive value of depressive symptoms is important knowledge for clinical practice and highlights the process of “adverse selection”. Adverse selection is a term coined to indicate the paradox where patients with mental health symptoms are known to have an increased risk for opioid misuse but unfortunately are also those who receive more opioids [20]. A greater awareness of and intervention on patients’ pain psychology may mitigate this risk. For example, while there were no significant mean level differences between psychological variables between those who did and did not receive the IMMPR, there were different statistically significant psychological predictors of future opioid outcomes between these groups.

While direct comparisons should be done cautiously, it is of value to note that there were no significant associations between pain intensity and depressive symptoms and opioid outcomes in the sample participating in IMMRP while these associations were consistently significant in the non-IMMRP sample. For both groups, there were significant univariate associations between dysfunctional pain coping and different operationalizations of opioid outcomes and in the multivariate analysis predicting LTOT, dysfunctional pain coping was a unique predictor in the subsample participating in IMMRP, after controlling for prescription history. One possibility is that IMMRP participation may have influenced the associations between pain, depressive symptoms, dysfunctional pain coping and risk for LTOT. Possibly, the treatment program influenced depressive symptoms and pain (or their association). Unfortunately, this study does not contain follow up data on IMMRP outcome that can support this hypothesis, but future studies using the SRPQ registry could study this.

In the IMMRP sample, there was a significant association between widespread pain and LTOT, indicating a decreased likelihood of future LTOT for those who have widespread pain. However, this association did not retain significance after controlling for opioid prescription history. This suggests that those with widespread pain were less likely to receive opioids before as well as after IMMRP. This could be related to a following of guidelines within this subgroup, as these highlight the lack of efficacy for opioid treatment for fibromyalgia where widespread pain is a cardinal symptom [50].

There were no significant correlations between LTOT and demographic variables in either of the samples. The same was true for correlations between anxiety and any of the opioid outcomes. Nor was interpersonally distressed coping found to be of high relevance for opioid outcomes, showing no significant associations with future strong opioid prescriptions or LTOT. There was however an inverse association between interpersonally distressed coping and any opioid prescriptions, suggesting that patients expressing more interpersonally distressed coping when it comes to management of their pain condition were less likely to be prescribed an opioid. Possibly but speculatively, interpersonally distressed patients elaborate more on their psychosocial difficulties during the clinical encounter, which has been found to be inversely related to the likelihood of a doctor initiating a biomedical intervention [47].

### 4.2. Limitations

In this study there are limitations, first regarding those excluded (18%) due to missing data. Excluded participants differed from the included participants in some but not most variables. Specifically, those not included in the analyses had a lower level of education, had less spread pain, a slightly higher level of depressive symptoms, were more likely to be men, have a history of opioid prescription and have participated in the IMMRP. Although these differences were mostly small, suggesting that any bias may not be large, loss of participants due to missing data may have affected the internal and external validity of the results.

Second, there are also some limitations related to sample selection and power. The sample in this study consists of patients from two university clinics in Sweden. Even if there are differences between pain rehabilitation clinics in Sweden, the patients in this study can be said to generally represent the study population of patients with chronic musculoskeletal pain in specialist care Sweden [51]. The number of participants is big enough for most of the analysis in the study but the analysis of LTOT could suffer from insufficient power due to small groups.

Third, choices of how opioid use was operationalized may have influenced results. While we follow previous research [13], the demarcation between weak and strong opioids is based on previously held assumptions that the risk of abuse and overconsumption is lower in weak opioids, which is questioned today [52]. In addition, the operationalization of opioid use was inferred from the prescriptions dispensed from pharmacies, but it should be noted that we do not know whether participants took the medications they were dispensed. Even though the number of patients with widespread pain was known, indicating a subgroup of nociplastic pain we do not know how the chronic pain conditions were classified, e.g., the proportion of neuropathic and nociceptive pain.

Fourth, there are limitations regarding measurement of the pain coping profiles. The use of coping profiles using the MPI incorporates a range of biopsychosocial characteristics and provides little detail on the specific pain psychological mechanisms that may be relevant for understanding the pain problem. For example, measures of pain acceptance, pain catastrophizing or pain related fear would have added detail to the understanding of possible mechanisms that may link pain psychological aspects to opioid use.

Finally, there are limitations regarding study design. This is an observation study, meaning we cannot draw any conclusions on causal processes explaining the associations.

## 5. Conclusions

This study shows that opioid treatment is common among patients with chronic pain seeking care in specialized pain rehabilitation clinics. It also indicates associations between individual characteristics and opioid prescriptions in Swedish pain rehabilitation, specifically pain and depressive symptoms. Understanding how individual characteristics interrelate and relate to prescription patterns and long-term opioid use is a prerequisite for better management and fundamental for preventing overuse.

## Figures and Tables

**Table 1:** Opioids prescribed to the total sample (*N* = 1334).

Opioid Medications	ATC	Prescriptions—*N*	Prescriptions—%
Morphine	N02AA01	679	7.5
Oxycodone	N02AA05	1027	11.3
Oxycodone/Naloxone	N02AA55	66	0.7
Ketobemidone	N02AB01	94	1
Fentanyl	N02AB03	348	3.8
Dextropropoxyfen	N02AC04	153	1.7
Buprenorphine	N02AE01	330	3.6
Morphine/antispasmodic	N02AG01	182	2
Ketobemidone/antispasmodic	N02AG02	10	0.1
Codeine/paracetamol	N02AJ06	1805	19.8
Codeine/ibuprophene	N02AJ08	29	0.3
Codeine other comb	N02AJ09	149	1.6
Tramadol	N02AX02	4173	45.9
Tapentadol	N02AX06	55	0.6
Total		9100	100

ATC, Anatomical Therapeutic Chemical.

**Table 2:** Characteristics of the two samples.

Baseline	IMMRP Participants *N* = 321	Non-IMMRP Participants *N* = 1017	t/χ^2^
Age	38.7(9.9)	41.6 (11.6)	4.10 **
Sex (% women)	84%	70%	23.29 **
Education (% post-secondary)	24%	22%	0.54
Pain intensity (0–10)	6.8 (1.8)	7.0 (1.7)	1.82
Pain spreading (0–36)	14.6 (7.5)	13.9 (8.3)	−1.35
Interpersonally distressed coping (0–100)	29.2 (38.7)	24.6 (36.9)	−1.92
Dysfunctional coping (0–100)	43.9 (41.5)	44.1 (42.6)	0.055
Anxiety symptoms (0–21)	8.3 (4.3)	8.4 (5.0)	0.17
Depressive symptoms (0–21)	8.2 (3.9)	8.1 (4,6)	−0.437
Any opioids one year prior (% yes)	37%	39%	0.34
Strong opioids one year prior (% yes)	3.1%	8.1%	9.41 **
Follow up two years after assessment			
Any opioids (% yes)	55.1%	54.7%	0.02
Strong opioids (% yes)	12.5%	20.0%	9.40 *
LTOT (% yes)	3.1%	6.6%	5.49 *

* = *p* < 0.05; ** = *p* < 0.01.

**Table 3:** Univariate associations between baseline predictors and opioid outcomes for the sample who did, and did not, receive IMMRP after multidisciplinary assessment.

		Any Opioids Two Years After	Strong Opioids Two Years After	LTOT Two Years After
IMMRP participants	Age	−0.10	0.004	0.02
	Sex	0.03	0.12 *	0.07
	Education	−0.15^**^	−0.08	−0.02
	Pain intensity	0.04	0.02	−0.02
	Pain spreading	−0.16 **	−0.07	−0.12 *
	Interpersonally distressed coping	−0.17 **	−0.08	−0.06
	Dysfunctional coping	0.19 **	0.12 *	0.11 *
	Anxiety symptoms	0.03	0.07	0.003
	Depressive symptoms	0.07	0.10	0.05
	Opioids 1 year prior (any)	0.34 **	0.06	0.12 *
	Opioids 1 year prior (strong)	0.09	0.31 **	0.38 **
Non-IMMRP participants	Age	0.03	0.11 **	0.05
	Sex	0.03	0.06	0.04
	Education	−0.06 *	0.01	−0.01
	Pain intensity	0.14 **	0.13 **	0.16 **
	Pain spreading	0.08 *	−0.01	0.05
	Interpersonally distressed coping	−0.07 *	−0.04	−0.04
	Dysfunctional coping	0.18 **	0.13 **	0.13 **
	Anxiety symptoms	0.04	0.03	0.06
	Depressive symptoms	0.17 **	0.13 **	0.14 **
	Opioids 1 year prior (any)	0.36 **	0.23 **	0.23 **
	Opioids 1 year prior (strong)	0.15 **	0.29 **	0.33 **

Note. Depending on variable characteristics, point-biserial (correlations between continuous and dichotomous variables) or Phi (correlations between two dichotomous variables) were used. * = *p* < 0.05; ** = *p* < 0.01.

**Table 4:** Prediction of LTOT in the sample who did participate in IMMRP (*N* = 321).

				Controlling for Strong Opioids One Year Before		
Predictors	OR	95% CI	*p*-Value	OR	95% CI	*p*-Value
Pain spreading	0.89	0.79–0.99	0.04 *	0.89	0.76–1.02	0.07
Dysfunctional coping	1.02	0.998–1.03	0.09	1.02	1.00–1.05	0.04 *
Strong opioids one year prior				56.85	8.12–398.03	0.0001 **

* = *p* < *0*.05; ** = *p* < *0*.01.

**Table 5:** Prediction of LTOT in the sample who did not participate in IMMRP (*N* = 1013).

				Controlling for Strong Opioids One Year Before		
Predictors	OR	95% CI	*p*-Value	OR	95% CI	*p*-Value
Pain intensity	1.83	1.23–2.71	0.003 *	1.62	1.06–2.48	0.03 *
Dysfunctional coping	1.01	0.999–1.01	0.12	1.01	0.999–1.01	0.07
Depressive symptoms	1.07	1.01–1.13	0.02 *	1.09	1.02–1.16	0.008 **
Strong opioids one year prior				11.08	6.03–20.36	0.0001 **

* = *p* < *0*.05; ** = *p* < *0*.01.

## Data Availability

The datasets generated and/or analyzed in this study are not publicly available as the Ethical Review Board has not approved the public availability of these data.

## Supplementary Materials

The following are available online at https://www.mdpi.com/article/10.3390/jcm10102130/s1, Table S1: Anatomical Therapeutic Chemical (ATC) codes for in- and excluded medications.
