# Supplementary material for: Opioid Prescriptions in Chronic Pain Rehabilitation. A Prospective Study on the Prevalence and Association between Individual Patient Characteristics and Opioids
Source: J Clin Med. 2021 May 14;10(10):2130. doi: 10.3390/jcm10102130 (PMC8155870; doi:10.3390/jcm10102130)
Supplement: Supplementary file 1 [file jcm-10-02130-s001.zip › jcm-1188292-proofed-supplementary.pdf]

Table S1. Anatomical Therapeutic Chemical (ATC) codes for in- and excluded medications.

**Opioid drugs**	**ATC**	**Weak or strong classification**
Included medications		
Morphine	N02AA01	Strong
Oxycodone	N02AA05	Strong
Oxycodone/Naloxone	N02AA55	Strong
Ketobemidone	N02AB01	Strong
Fentanyl	N02AB03	Strong
Dextrtopropoxyfen	N02AC04	Weak
Buprenorphine	N02AE01	Strong
Morphine/antispasmodic	N02AG01	Strong
Ketobemidone/antispasmodic	N02AG02	Strong
Codeine/paracetamol	N02AJ06	Weak
Codeine/ibuprophene	N02AJ08	Weak
Codeine other comb	N02AJ09	Weak
Tramadol	N02AX02	Weak
Tapentadol	N02AX06	Strong
Excluded medications		
Hydromorphone	N02AA03	Strong
Dextrtopropoxyfen comb	N02AC54	Weak
Buprenorphine	N02BC01	Strong
Methadone	N02BC02	Strong
**Buprenorphine comb**	**N02BC51**	**Strong**
